# Supplementary material for: Patterns of Intron Gain and Loss in Fungi
Source: PLoS Biol. 2004 Nov 30;2(12):e422. doi: 10.1371/journal.pbio.0020422 (PMC532390; doi:10.1371/journal.pbio.0020422)
Supplement: Table S1 — Also available at http://genes.mit.edu/NielsenEtAl/. (4.3 MB ZIP). [file pbio.0020422.st001.zip › NielsenEtAl/html/871.html]

AN7042.1.NCU06587.1.MG01707.1.FG05267.1


```
 CLUSTAL W (1.82) Multiple Sequence Alignments - Introns Inserted


Sequence 1: NCU06587.1	327 aa
Sequence 2: FG05267.1	281 aa
Sequence 3: MG01707.1	296 aa
Sequence 4: AN7042.1	360 aa
Alignment Length: 397 aa
Number Identitical Residues: 120 aa
Alignment Score (without introns) 5984


MG01707.1 	MWWSNSNKEPEKSPKAEAASSSSKDTAPTKDGAS-----------KAFDPNSLPERQKLK
NCU06587.1	MWPFGNSNNPD---KPAPPTQSPSETSPIQDKVEKKVVDVKNAVHDELNPQKLPARRKLP
FG05267.1 	MW-WGKSKDP----KPE----EKSEVAQIKDTVV----------------EKLPSKEQIS
AN7042.1  	MFWGSRKEEPS----------EPPALPPVK-------------------------RGKLS
          	*:  . .::*.          .    .  :                         : :: 

MG01707.1 	PSLQKIVDQADREDNFYDQLVDGY2APESTESNVRYAGYAARLRTIMLSAHRYVAYTSDI
NCU06587.1	DGLQKLVDKTDKE-EFYDDLYEGY2MPPSTESNVRYAAYASRFRTILLSAHRYVAYTSDI
FG05267.1 	NKVQDSVNQSGKNGNVLNDIAEGY2GPDSTDSNLRYAAYATRIRTILLSAHRYVAYTSDI
AN7042.1  	PQLQKIVDSDDTN--PYDDVYSYY2SVDSTDTNYRYAGYATRLRTLLLSAHRYVAYTSDV
          	  :*. *:. . :    ::: . *    **::* ***.**:*:**::************:

MG01707.1 	GESFRPVAHPYLVKGAYGVSWLYILGDVSHEGYKAYWQNQRTRNPQVALT------AHQQ
NCU06587.1	GESFRPVAHPWLVRGAYGVSWAYILGDVSYEGYKAYWHNRRVLDPNVELT------SRQK
FG05267.1 	GESFRPVAHPNLVRGAYGVSWLYLIGDVSYEGYKSYWHNQRVLNPTLQLS------PRQE
AN7042.1  	GESFRPVAHPILVRSAYAVSWAYLLGDVGHEGYKAYLRNRRVLAPPCEAYKDSRDLTHQQ
          	********** **:.**.*** *::***.:****:* :*:*.  *     ..: . .:*:

MG01707.1 	RILGSDAT----------------SEHSTKLEPG---VVTPLEDYRTVMAQRAIFQSLAS
NCU06587.1	RILGLIDENG------------TKKMEDDKPTPG---KITPLEDYRTVMLQRLIFQSVAS
FG05267.1 	KITGLP------------------ALETRAIEPG---TVAPLDDYRTVMVQRAIFQSVAS
AN7042.1  	IVMGMATGNVGGSLSSADWGEGQKQVDSDTLTPWPTAHIPLIEDYRVVMAKRAVFQSIAS
          	 : *    . ..: :::. ... .  .     * .:: :. ::***.** :* :***:**

MG01707.1 	MGLPAFTIHSVVRYSGRALKEVKNKNIRTWAPIG~LGLAVVPFLPSLFDKPVEDAVEWTF
NCU06587.1	MGLPAFTIHSVVRYSGRAMKNVKNQTIRTWGPIG~LGLSVVPFLPTLFDKPVENAVEWVF
FG05267.1 	MGLPAFTIHSVVRYSGRAMKDMKNKVIRTWGPIG~LGLAVVPFLPAMFDEPVENAVEWAF
AN7042.1  	MGLPALTIHSVVRYSGRALKGAKSTFARTWVPIG0LGLSVVPFLPYIFDHPVDEAVDWTF
          	*****:************:*  *.   *** *** ***:****** :**.**::**:*.*

MG01707.1 	HKAFATVGGKE2TVG----DTPATG----------------------------------R
NCU06587.1	HKAFETFGGKE~AVG----HAPLIG----------------------------------R
FG05267.1 	HKGFETFGGKG~AVG----NAPMTG----------------------------------R
AN7042.1  	RTGLRIYGGED~AVKPLPQHPHAAGGTTHEEDSTSLSHFHIKAPEVADAASANVSWDEYK
          	:..:   **:  :* . ....   *.:: ...::: :    .:.. :.::::. : .. :

MG01707.1 	ERQLNEK-VKEKEL--~------------------------
NCU06587.1	EKTLSEKPTPHKEKNE2KSPN-IFPVPALGIRKCP------
FG05267.1 	EDLLAKKPVKEKEL--~------------------------
AN7042.1  	EELRRAKERRKREREE~RGESGILAMLGFGSKQKEEKGKTE
          	*     *   .:* ..  . ..  .  . .  .  ....:.
```
